# Selective electrochemical production of hydrogen peroxide at zigzag edges of exfoliated molybdenum telluride nanoflakes

**DOI:** 10.1093/nsr/nwaa084

**Published:** 2020-04-25

**Authors:** Xuan Zhao, Yu Wang, Yunli Da, Xinxia Wang, Tingting Wang, Mingquan Xu, Xiaoyun He, Wu Zhou, Yafei Li, Jonathan N Coleman, Yanguang Li

**Affiliations:** Institute of Functional Nano and Soft Materials (FUNSOM), Jiangsu Key Laboratory for Carbon-Based Functional Materials and Devices, Soochow University, Suzhou 215123, China; College of Chemistry and Materials Science, Nanjing Normal University, Nanjing 210023, China; College of Material Science and Opto-Electronic Technology, University of Chinese Academy of Sciences, Beijing 100049, China; Institute of Functional Nano and Soft Materials (FUNSOM), Jiangsu Key Laboratory for Carbon-Based Functional Materials and Devices, Soochow University, Suzhou 215123, China; Institute of Functional Nano and Soft Materials (FUNSOM), Jiangsu Key Laboratory for Carbon-Based Functional Materials and Devices, Soochow University, Suzhou 215123, China; School of Physical Sciences and CAS Key Laboratory of Vacuum Sciences, University of Chinese Academy of Sciences, Beijing 100049, China; School of Physics, CRANN and AMBER Centers, Trinity College Dublin, Dublin 2, Ireland; School of Physical Sciences and CAS Key Laboratory of Vacuum Sciences, University of Chinese Academy of Sciences, Beijing 100049, China; College of Chemistry and Materials Science, Nanjing Normal University, Nanjing 210023, China; School of Physics, CRANN and AMBER Centers, Trinity College Dublin, Dublin 2, Ireland; Institute of Functional Nano and Soft Materials (FUNSOM), Jiangsu Key Laboratory for Carbon-Based Functional Materials and Devices, Soochow University, Suzhou 215123, China

**Keywords:** hydrogen peroxide production, non-precious-metal-based electrocatalyst, molybdenum telluride, liquid phase exfoliation, zigzag edges

## Abstract

The two-electron reduction of molecular oxygen represents an effective strategy to enable the green, mild and on-demand synthesis of hydrogen peroxide. Its practical viability, however, hinges on the development of advanced electrocatalysts, preferably composed of non-precious elements, to selectively expedite this reaction, particularly in acidic medium. Our study here introduces 2H-MoTe_2_ for the first time as the efficient non-precious-metal-based electrocatalyst for the electrochemical production of hydrogen peroxide in acids. We show that exfoliated 2H-MoTe_2_ nanoflakes have high activity (onset overpotential ∼140 mV and large mass activity of 27 A g^−1^ at 0.4 V versus reversible hydrogen electrode), great selectivity (H_2_O_2_ percentage up to 93%) and decent stability in 0.5 M H_2_SO_4_. Theoretical simulations evidence that the high activity and selectivity of 2H-MoTe_2_ arise from the proper binding energies of HOO^*^ and O^*^ at its zigzag edges that jointly favor the two-electron reduction instead of the four-electron reduction of molecular oxygen.

## Teaser

2H-MoTe_2_ nanoflake prepared from liquid phase exfoliation exhibit remarkable electrocatalystic activity for oxygen reduction to hydrogen peroxide in acids.

## INTRODUCTION

Hydrogen peroxide (H_2_O_2_) is a potential energy carrier and an important commodity chemical with high industrial value [1,2]. Its low-concentration (3–9 wt%) solution is also widely used for a vast range of environmental, medical and household applications. At present, over 99% of H_2_O_2_ is produced via the energy-intensive anthraquinone oxidation process [3,4]. For economic reasons, this process only operates in centralized reactors on a large scale, and produces highly concentrated H_2_O_2_ that often has to be distributed to, and diluted at, the site of use, bringing additional complexity and challenges [5]. In addition to the anthraquinone oxidation process, H_2_O_2_ can also be directly converted from H_2_ and O_2_ in the presence of Pd-based catalysts [4,6]. The practical viability of this high-pressure conversion, however, is seriously undermined by its potential hazard of explosion. There is a pressing call to explore other effective methods for the green, mild and on-demand production of H_2_O_2_.

The electrochemical synthesis of H_2_O_2_ from oxygen reduction reaction (ORR) represents an ideal solution. ORR can take place via a four-electron pathway or a two-electron one. The former yields water as the reduction product, and is the key reaction at the cathode of fuel cells and aqueous metal-air batteries [7–9]. The latter enables the direct production of H_2_O_2_ at ambient conditions, but was substantially less investigated until very recently [5,10]. Due to the competitive nature of these two pathways, suitable electrocatalysts are required to selectively promote the two-electron ORR (2e-ORR) process. Previous theoretical study established the binding energy of HOO^*^ (Δ*G*_HOO^*^_) on catalyst surface as the activity descriptor, with the highest activity achieved at the optimal Δ*G*_HOO^*^_ ∼ 4.2 eV [11]. This selection rule was used to guide the computational screening of new alloy electrocatalysts and validated through experiments. At present, the state-of-the-art 2e-ORR electrocatalysts are Pt-Hg and Pd-Hg alloys [11,12], followed by Au alloys [13–15]. Despite their relatively high mass activity and selectivity in acids, these precious metal alloys are unlikely to be used on a large scale due to their prohibitive costs and sometimes high toxicity. On the other hand, carbon-based materials (e.g. oxidized carbon nanotubes and reduced graphene oxide) exhibit appreciable 2e-ORR activity and selectivity in alkaline solution but generally very poor performance in neutral or acidic solution [16–18]. Their potentials are also limited since H_2_O_2_ is subjected to rapid decomposition in alkaline medium. We are therefore in great need of developing non-precious-metal-based electrocatalysts with outstanding selectivity and activity for 2e-ORR in acids.

Over recent years, two-dimensional (2D) transition metal dichalcogenides (TMDs) have attracted intense research interest owing to their unique anisotropic structures and intriguing physical and chemical properties [19–21]. Many of them (as best exemplified by MoS_2_) are well known to be active for electrocatalytic hydrogen evolution reaction (HER) [22–24]. Computational and experimental studies suggest that their active sites are often located at the edges [25], and that the catalytic activities can be tuned via proper alloying, doping, and defect and strain engineering [26–29]. However, the potential of TMDs for other electrocatalytic reactions beyond HER remains to be explored. In this study, we demonstrate that 2H-phase molybdenum telluride (MoTe_2_) nanoflakes, synthesized from bulk powder via ultrasonication-assisted liquid phase exfoliation, acts as an efficient 2e-ORR electrocatalyst in acids. They are measured to catalyze the electrochemical production of H_2_O_2_ with high activity, selectivity and stability. Our theoretical calculations reveal that the high activity and selectivity can be attributed to the favorable binding of HOO^*^ and weak binding of O^*^ at the zigzag edges.

## RESULTS AND DISCUSSION

### Exfoliation and characterizations of MoTe_2_ nanoflakes

**Figure 1.**
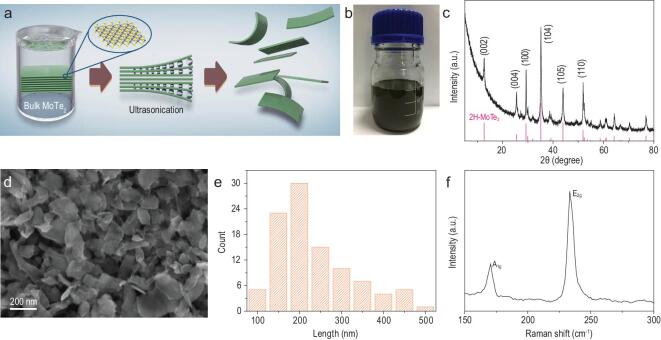
Preparation and structural characterizations of MoTe_2_ nanoflakes. (a) Schematic exfoliation process of bulk MoTe_2_ powders to nanoflakes by LPE. (b) A photo showing the dispersion of MoTe_2_ nanoflakes in ethanol. (c) XRD and (d) SEM images of MoTe_2_ nanoflakes. (e) Histogram of lateral size distribution of MoTe_2_ nanoflakes estimated from multiple SEM images. (f) Raman spectrum of MoTe_2_ nanoflakes.

Even though synthesis of high-quality MoTe_2_ nanosheets or nanoflakes by chemical vapor deposition (CVD) has been reported in literature [30,31], such a high-temperature bottom-up approach is seriously limited by its complexity and low production yield. Here, top-down liquid phase exfoliation (LPE) was employed for its notable simplicity, scalability and reproducibility [32,33]. Commercial crystalline MoTe_2_ powders were ultrasonicated in N-methylpyrrolidone (NMP) (see Experimental Method in the Supplementary data for details). During the ultrasonication, the weak interlayer van der Waals interactions were interrupted; the 2D bulk crystal was then exfoliated to few-layered nanoflakes stabilized by NMP (Fig. 1a). These nanoflakes were collected by centrifugation and could be readily re-dispersed in common solvents. Figure 1b showed exfoliated MoTe_2_ nanoflakes re-dispersed in ethanol. The dispersion was stable for weeks without obvious sediment. Powder X-ray diffraction (XRD) pattern of the product confirmed that it was composed of hexagonal 2H MoTe_2_, which is the most stable form of MoTe_2_ at low temperatures. Under scanning electron microscopy (SEM), the product was revealed to consist of nanoflakes (Fig. 1d and e). Additional SEM image and low-magnification transmission electron microscopy (TEM) images were supplemented in Fig. S1. Based on TEM statistics, the lateral size of MoTe_2_ nanoflakes was analyzed to be in the range of 50–350 nm with the mean length ∼143 nm (Fig. S1). Raman spectrum of the product displayed two pronounced peaks at 170 and 233 cm^−1^ (Fig. 1f), which are characteristic to the A_1g_ and E_2g_ modes of 2H MoTe_2_, respectively [34,35].

**Figure 2.**
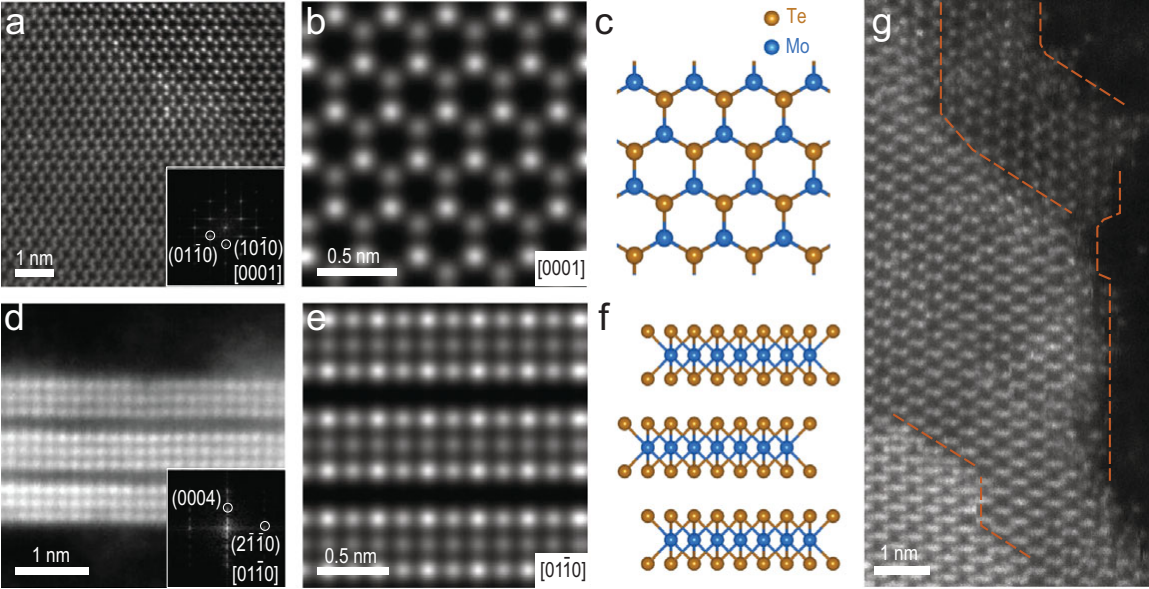
STEM characterization of MoTe_2_ nanoflakes. (a) STEM ADF image of multilayer MoTe_2_ along the *c*-axis and the corresponding FFT pattern (inset). (b) Simulated ADF image of tri-layer 2H MoTe_2_ along the *c*-axis. (c) Structural model of 2H MoTe_2_ along the *c*-axis. (d) STEM ADF image of MoTe_2_ along the *b*-axis and the corresponding FFT pattern (inset). (e) Simulated ADF image of 2H MoTe_2_ along the *b*-axis. (f) Structural model of 2H MoTe_2_ along the *b*-axis. (g) Edge structures of MoTe_2_ nanoflakes; the dashed orange lines highlight the zigzag edges.

To elucidate the atomic structure of exfoliated MoTe_2_ nanoflakes, aberration-corrected scanning transmission electron microscopy (STEM) was utilized. Figure 2a showed a typical annular dark field (ADF) image of the product, which could be identified as 2H MoTe_2_ along the *c*-axis based on the honeycomb pattern of the atomic sites and the corresponding fast Fourier transform (FFT) pattern. The assignment was further corroborated by the excellent agreement between the experimental image and the simulated ADF image of 2H MoTe_2_ (0001) plane (Fig. 2b and c). Cross-sectional STEM-ADF image of MoTe_2_ nanoflakes was also acquired as shown in Fig. 2d. Its corresponding FFT pattern matched with the diffraction pattern of 2H MoTe_2_ along the [$01\bar{1}0$] zone axis. The simulated ADF image along this zone axis also agreed well with the experimental image (Fig. 2e and f). Furthermore, since the intensity of STEM-ADF image increased approximately linearly with the number of layers in thin 2D crystals, we could determine the nanoflake thickness for up to seven layers (Fig. S2) and directly examine the stacking mode between layers by quantifying the image intensity. As summarized in Fig. S3, the Mo and Te_2_ atoms in the monolayer honeycomb lattice of H-MoTe_2_ exhibited very different ADF image intensities, while the atomic sites in the bilayer honeycomb lattice showed very similar image intensity, a characteristic feature for 2H stacking with overlapped Mo+Te_2_ sites. This intrinsic feature of 2H-stacking MoTe_2_, with 180° rotation between two adjacent layers, could help us identify the edge orientations in multilayer flakes. The exposed edges, though not atomically sharp, were mostly along the zigzag directions with abundant unsaturated Mo and Te bonds (Fig. 2g), which might be responsible for the observed high electrocatalytic activity as described in the following part [36].

### Electrochemical production of H_2_O_2_

**Figure 3.**
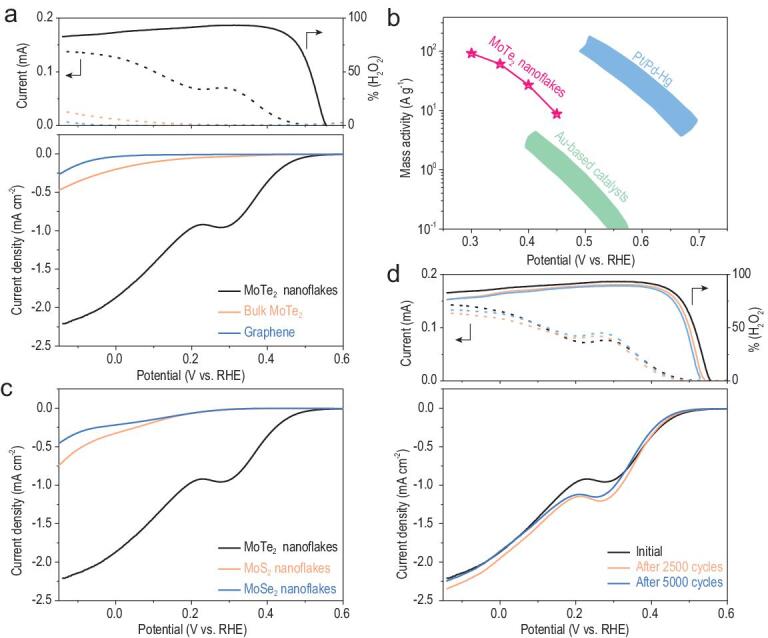
Electrochemical performance of MoTe_2_ nanoflakes. (a) (Lower panel) polarization curves of MoTe_2_ nanoflakes, bulk MoTe_2_ powders and graphene nanosheets alone, respectively and (upper panel) the corresponding ring currents (dashed line) and H_2_O_2_ percentage (solid line). (b) Derived mass activity of MoTe_2_ nanoflakes in comparison with those of Pt/Pd-Hg alloys [11,12] and Au-based catalysts [14,15] estimated from literature. (c) Polarization curves of MoTe_2_, MoS_2_ and MoSe_2_ nanoflakes, respectively. (d) Polarization curves, ring currents and H_2_O_2_ percentage of MoTe_2_ nanoflakes at the initial state and after certain numbers of cycles during the accelerated durability test. The electrolyte in use was O_2_-saturated 0.5 M H_2_SO_4_; the electrode rotating speed was 1600 rpm.

To assess the electrocatalytic performance of MoTe_2_ nanoflakes for 2e-ORR to H_2_O_2_, they were physically mixed with graphene nanosheets exfoliated from graphite powders as the conductive additive (Fig. S4, see Experimental Method in the Supplementary data for details). Graphene nanosheets were used here instead of conventional carbon black because of their superior electrical conductivity and similar 2D geometry that could form better contacts with MoTe_2_ nanoflakes [37]. The electrocatalyst mixture was then loaded onto the glassy carbon disk of a rotating ring disk electrode (RRDE) with an active material loading of 10 μg cm^−2^. RRDE voltammograms were separately carried out in N_2_-saturated and O_2_-saturated 0.5 M H_2_SO_4_ at the electrode rotating speed of 1600 rpm (Fig. S5). Corresponding ORR polarization curves were then derived from their differences, and summarized in the lower panel of Fig. 3a. Exfoliated graphene nanosheets alone were electrochemically inert and exhibited no apparent cathodic current density till at <0 V (versus reversible hydrogen electrode or RHE, the same hereafter). Bulk MoTe_2_ mixed with graphene nanosheets also had a negligible activity. By stark contrast, exfoliated MoTe_2_ nanoflakes demonstrated a dramatically improved performance with an onset potential as positive as ∼0.56 V (corresponding to an overpotential of ∼140 mV), close to the state-of-the-art PtHg_4_ alloy reported in literature (∼0.6 V) [11]. Their cathodic current density continuously increased beyond the onset, and reached ∼1.9 mA cm^−2^ at 0 V. The broad wave centered at ∼0.3 V was likely associated with the reduction of the surface oxide. Based on the concurrently measured ring current, we further derived the H_2_O_2_ percentage in the product from MoTe_2_ nanoflakes (upper panel of Fig. 3a). It was shown to stay >80% at almost the entire potential region with a recorded peak value of ∼93%. Such remarkable selectivity was also comparable to Pt-Hg and Pd-Hg alloys [11,12]. In addition, the effect of catalyst loading on the geometric current density and H_2_O_2_ percentage was investigated. Increasing the loading was found to slightly enlarge the cathodic current density but adversely compromise the reaction selectivity (Fig. S6). We speculated that the higher catalyst loading helped retain produced H_2_O_2_, and caused it to be further reduced to H_2_O via another two-electron pathway, therefore giving rise to lower H_2_O_2_ selectivity.

In order to allow direct activity comparison with other reported 2e-ORR electrocatalysts in acids, we normalized the H_2_O_2_ partial current of MoTe_2_ nanoflakes over the mass of active material as presented in Fig. 3b. The mass activity was calculated to be in the range of ∼10–10^2^ A g^−1^ between 0.3–0.45 V, which, although still not as magnificent as the state-of-the-art Pt-Hg and Pd-Hg alloys [11,12], was superior to Au alloys [14,15] and carbon-based materials [38,39]. For example, the mass activity of MoTe_2_ nanosheets at 0.4 V was 27 A g^−1^—which is ∼7–10 times greater than those of Au-Pd alloys [14,15] and N-doped carbon [38,39]. Interestingly, MoTe_2_ seemed to be quite unique among TMD materials for the electrochemical production of H_2_O_2_. Its sulfide and selenide analogues—MoS_2_ and MoSe_2_ nanoflakes likewise exfoliated from corresponding bulk powders and hybridized with graphene nanosheets—exhibited significantly worse activities (Fig. 3c).

Stability is another key parameter in the electrocatalyst assessment. Despite the concern over the susceptibility of MoTe_2_ to oxidation, we found that exfoliated MoTe_2_ nanoflakes had very decent electrochemical stability under the 2e-ORR working condition, at least for several days. To demonstrate this, our electrocatalyst was subjected to an accelerated durability test by rapidly cycling between 0 and 0.3 V at 100 mV s^−1^ in O_2_-saturated electrolyte for a predetermined number of cycles, and then measuring its polarization curves. Figure 3d compared the polarization curves at the initial state, after 2500 cycles and after 5000 cycles. The measured disk polarization curves remained largely similar except for the reduction wave at ∼0.3 V presumably due to the partial surface oxidation during the durability test. There was only a slight decrease in the potential-dependent H_2_O_2_ percentage likely as a result of the catalyst oxidation. Moreover, even after MoTe_2_ nanoflakes were aged overnight (>12 h) in the O_2_-saturated electrolyte at the open-circuit potential, only slight current decay occurred and no apparent selectivity decay were observed as compared to the fresh electrode (Fig. S7).

### DFT calculations

**Figure 4.**
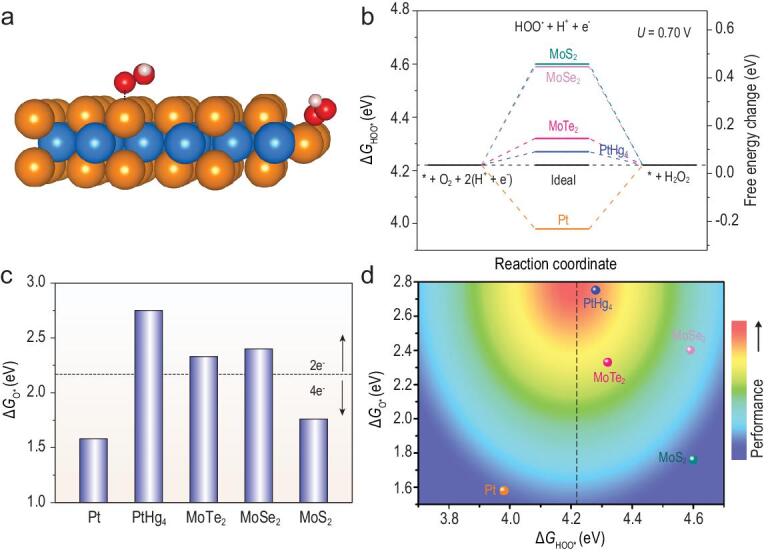
DFT simulations of the 2e-ORR pathway on 2H MoTe_2_. (a) Optimized structure of HOO^*^ adsorbed on the basal plane or edge site; blue, orange, red and white spheres represent Mo, Te, O and H atoms, respectively. (b) Δ*G*_HOO^*^_ for 2e-ORR to H_2_O_2_ on Pt [11], PtHg_4_ [11], MoTe_2_, MoSe_2_ and MoS_2_ at the equilibrium potential of *U*_O2/H2O2_ = 0.70 V and the corresponding free-energy profiles. (c) Δ*G*_O^*^_ of Pt [11], PtHg_4_ [11], MoTe_2_, MoSe_2_ and MoS_2_; the position of the dashed line (2.17 eV) denotes the average value of Δ*G*_O^*^_ on Pt (typical 4e-ORR electrocatalyst) and PtHg_4_ (typical 2e-ORR electrocatalyst), which was proposed to be the boundary between 4e and 2e selectivity. (d) 2D ‘heat map’ for 2e-ORR performance (both activity and selectivity); the dashed line represents the optimal Δ*G*_HOO^*^_ for H_2_O_2_ production.

In order to understand the origin of the high activity and selectivity of MoTe_2_ nanoflakes, spin-unrestricted density functional theory (DFT) calculations were carried out to simulate the 2e-ORR process on both the basal plane and edge site of 2H MoTe_2_ as well as several other materials. The basal plane slab was modeled using a 4 × 4 × 1 supercell (Fig. S8). Previous experimental studies on MoS_2_ demonstrated that its most stable edges were zigzag-type with the Mo atoms covered by 50% S coverage [40,41]. As a result, the Mo-edge slab in our study was constructed with 50% Te coverage and a periodicity of 3 Mo atoms, which was determined to be the most stable configuration (Fig. S9). 2e-ORR to H_2_O_2_ generally involves two elemental steps: molecular O_2_ is first transformed to HOO^*^ via a proton-coupled electron transfer, followed by the protonation and reduction of HOO^*^ to yield H_2_O_2_. Previous study established that the binding energy of HOO^*^ (Δ*G*_HOO^*^_) was an effective activity descriptor, and its optimal value was found to be ∼4.2 eV [42]. Following this guiding principle, Δ*G*_HOO^*^_ on different surface sites was computed and compared. Figure 4a showed the optimized structures of HOO^*^ adsorbed on the basal plane or edge of 2H MoTe_2_. Δ*G*_HOO^*^_ calculations suggested that the H_2_O_2_ formation was overwhelmingly challenging on the basal plane (Δ*G*_HOO^*^_ = 5.34 eV). Introduction of Te vacancies to the basal plane was also not helpful. For example, HOO^*^ could not even be produced over the single Te vacancy on the basal plane as it would be spontaneously dissociated to O^*^ adsorbed on the Te vacancy site and HO^*^ adsorbed on the nearby Te site (Fig. S10). By contrast, the reaction could readily proceed at the zigzag edge with Δ*G*_HOO^*^_ = 4.32 eV. Such an observation was interestingly reminiscent of the structure-dependent HER activities of 2H-MoS_2_ and many other TMD materials. Furthermore, the theoretical overpotential (*η*^t^) of 2e-ORR at the edge of MoTe_2_ was estimated by |Δ*G*_HOO^*^_/e − 4.22 V| and calculated to be 100 mV, which agreed reasonably well with the experimental value (∼140 mV), and was sufficiently close to that of PtHg_4_(110) surface (Δ*G*_HOO^*^_ = 4.28 eV and *η*^t^ = 60 mV) [11]. It unambiguously corroborated the high 2e-ORR activity of 2H MoTe_2_. Worth noting is that the armchair edge of MoTe_2_ (Fig. S11) was not catalytically active for 2e-ORR because it bound HOO^*^ too weakly (ΔG_HOO^*^_ = 4.60 eV). For the purpose of comparison, the HOO^*^ adsorption on Pt(111) was calculated to be considerably stronger than the ideal (Δ*G*_HOO^*^_ = 3.98 eV), and the HOO^*^ adsorption on MoS_2_ and MoSe_2_ edges significantly weaker than the ideal (Δ*G*_HOO^*^_ = 4.60 eV and 4.59 eV on MoS_2_ and MoSe_2_, respectively, Fig. 4b). The observed inefficiency of MoS_2_ and MoSe_2_ for 2e-ORR was therefore rationalized in spite of their structural similarity to MoTe_2_.

Of note, a favorable Δ*G*_HOO^*^_ value does not necessarily guarantee high H_2_O_2_ selectivity since 2e-ORR and 4e-ORR share the same initial step. The further transformation of HOO^*^ to O^*^ (4e-ORR pathway) would have to be effectively suppressed in order to achieve high H_2_O_2_ selectivity. As a result, weak O^*^ binding energy (Δ*G*_O^*^_) on the catalyst surface becomes a prerequisite. Based on this rationale, we could understand the predominant 4e-ORR selectivity on Pt owing to its very strong O^*^ affinity (Δ*G*_O^*^_ = 1.58 eV on Pt(111)), as well as the predominant 2e-ORR selectivity on PtHg_4_ owing to its very weak O^*^ affinity (Δ*G*_O^*^_ = 2.75 eV on PtHg_4_(110)) (Fig. 4c) [11]. Our calculations showed that the edge of 2H MoTe_2_ had Δ*G*_O^*^_ = 2.33 eV, which, albeit still not as positive as PtHg_4_, was sufficiently large to render MoTe_2_ H_2_O_2_-selective. It is also worth mentioning that MoS_2_ relatively favored 4e-ORR, and MoSe_2_ relatively favored 2e-ORR even though both of them had negligible activities. At last, based on the previous volcano for 2e-ORR built upon the sole activity descriptor Δ*G*_HOO^*^_ [11], we further compiled our computation results on a 2D heat map as shown in Fig. 4d. We implicitly assumed that the hottest spot was located at Δ*G*_HOO^*^_ = 4.22 eV and Δ*G*_O^*^_ = 2.75 eV. The Δ*G*_O^*^_ of PtHg_4_ (2.75 eV) was chosen as a reference given the fact that, in contrast to HOO^*^, there is not explicit criteria for denoting the optimal O^*^ affinity. According to this definition, the closer to the hottest spot, the better 2e-ORR performance, and vice versa. Among different materials under investigation, PtHg_4_ was in the region of the greatest activity and followed by MoTe_2_, while MoSe_2_, MoS_2_ and Pt were quite far away from the hot area.

## CONCLUSION

In summary, we for the first time introduced 2H-MoTe_2_ nanoflakes as a high-performance catalyst for electrochemical H_2_O_2_ production in acids. MoTe_2_ nanoflakes were exfoliated from the bulk powder via LPE in NMP. They were determined to have small thickness of a few nm, lateral sizes of 100∼500 nm and with preferentially exposed zigzag edges. When physically mixed with graphene nanosheets as the conductive additive, the MoTe_2_ nanoflakes demonstrated excellent activity and selectivity for 2e-ORR in 0.5 M H_2_SO_4_, with an onset potential at ∼0.56 V (*η* ∼140 mV), a large mass activity of 27 A g^−1^ at 0.4 V and high H_2_O_2_ selectivity up to ∼93%. Such a performance was far superior to those of Au alloys and N-doped carbon reported in literature, and approaching that of the state-of-the-art PtHg_4_ alloy. MoTe_2_ nanoflakes also exhibited impressive chemical and electrochemical stability in an accelerated durability test and overnight aging experiment. Finally, detailed DFT calculations showed that the high activity and selectivity of 2H MoTe_2_ originated from the favorable binding of HOO^*^ and weak binding of O^*^ at the zigzag edges, and thereby directly correlated the electrocatalytic performance with the unique anisotropic structure of MoTe_2_. Our study here unveiled the unexpected potential of MoTe_2_ nanoflakes as a non-precious-metal-based electrocatalyst for H_2_O_2_ production in acids, and might open a new pathway toward the catalyst design for this challenging electrochemical reaction.

## Supplementary Material

nwaa084_Supplemental_File
