# Supplementary material for: Selective electrochemical production of hydrogen peroxide at zigzag edges of exfoliated molybdenum telluride nanoflakes
Source: Natl Sci Rev. 2020 Apr 25;7(8):1360–6. doi: 10.1093/nsr/nwaa084 (PMC8288933; doi:10.1093/nsr/nwaa084)
Supplement: nwaa084_Supplemental_File [file nwaa084_supplemental_file.docx]

**Supplementary Data**

**Selective Electrochemical Production of Hydrogen Peroxide at Zigzag Edges of Exfoliated Molybdenum Telluride Nanoflakes**

Xuan Zhao^1†^, Yu Wang^2†^, Yunli Da^3†^, Xinxia Wang^1^, Tingting Wang^1^, Mingquan Xu^3^, Xiaoyun He^4^, Wu Zhou^3*^, Yafei Li^2*^, Jonathan N. Coleman^4^ and Yanguang Li^1*^

^1^Institute of Functional Nano and Soft Materials (FUNSOM), Jiangsu Key Laboratory for Carbon-Based Functional Materials and Devices, Soochow University, Suzhou 215123, China.

^2^College of Chemistry and Materials Science, Nanjing Normal University, Nanjing 210023, China.

^3^School of Physical Sciences and CAS Key Laboratory of Vacuum Sciences, University of Chinese Academy of Sciences, Beijing, 100049 China.

^4^School of Physics, CRANN and AMBER Centers, Trinity College Dublin, Dublin 2, Ireland.

**Corresponding Author**

*Y.G.L. E-mail: yanguang@suda.edu.cn

*Y.F.L. E-mail: liyafei@njnu.edu.cn

*W.Z. E-mail: wuzhou@ucas.ac.cn

†These authors contributed equally: X. Z., Y. W., Y. D.

**EXPERIMENTAL SECTION**

**Materials Preparation****.** MoTe_2_ nanoflakes were exfoliated by dispersing 1 g of commercial MoTe_2_ powders (from American Elements) in 50 mL of NMP, and ultra-sonicated with 6 s on and 2 s off pulses for 4 h using a horn-probe tip sonicator (Sonics Vibra-cell VCX-750W) operating at 80% amplitude. The dispersion was iced cooled during the sonication to avoid an obvious temperature rise. Unexfoliated MoTe_2_ powders were then isolated from the dispersion by centrifugation at 1500 rpm for 1.5 h, and were reusable for future exfoliation. The top dispersion was decanted and further centrifuged at 9000 rpm for 30 min. The sediment containing exfoliated MoTe_2_ nanoflakes was repetitively washed with ethanol and water, and finally lyophilized. MoS_2_ nanoflakes, MoSe_2_ nanoflakes and graphene nanosheets were similarly exfoliated from their corresponding bulk powders by LPE.

**Structural characterizations.** XRD was carried out on a PANalytical X-ray diffractometer (Cu Kα radiation). SEM image was obtained from a Zeiss Ultra 55 scanning electron microscope. Raman spectrum was collected from Horiba Jobin Yvon LabRAM HR800 with a laser excitation wavelength of 633 nm. Aberration-corrected STEM imaging was conducted on a Nion HERMES-100 microscope under the accelerating voltage of 60 kV. The convergence angle of probe-forming aperture was 32 mrad and the collection angle for ADF imaging was set to 75-210 mrad. MoTe_2_ nanoflakes were dispersed onto a microgrid with ultra-thin carbon film for their top-view images. Specimen for their cross-sectional imaging was prepared by ultramicrotomy after embedding the powder into resin. STEM-ADF image simulation was performed using QSTEM software.

**Electrochemical measurements****.** To prepare the working electrodes, 1 mg of exfoliated MoTe_2_ nanoflakes (or other catalyst powders) and 0.6 mg of exfoliated graphene nanosheets were dispersed in 2.5 mL of ethanol containing 60 µL of 5 wt% Nafion solution, and bath-sonicated for 40 min to form a homogeneous catalyst ink. A calculated aliquot (typically 6.5 µL) of the catalyst ink was then dropcast onto the glassy carbon disk (0.247 cm^2^) of a RRDE electrode and naturally dried to form a smooth catalyst film with an active material loading of typically 10 μg/cm^2^. Electrochemical experiments were carried out using a standard three-electrode system controlled by CHI 760 bipotentiostat. A saturated calomel electrode (SCE) was used as the reference electrode, and a graphite rod was used as the counter electrode. All the potential readings were measured against SCE and reported against RHE. No iR compensation was performed throughout our study. RRDE voltammetry in N_2_-saturated or O_2_-saturated 0.5 M H_2_SO_4_ was collected at 10 mV/s with the working electrode continuously rotating at 1600 rpm. To estimate the H_2_O_2_ yield from 2e-ORR, the potential of the Pt ring was held constant at 1.2 V. The H_2_O_2_ percentage in the product was determined from the following equation:

$${\%(H}_{2}O_{2})=200\cdot\frac{{I_{r}}/N}{I_{d}+{I_{r}}/N}$$

where *I_r_* was the ring current, *I_d_* was the disk current, and N (~ 0.37) was the calibrated current collection efficiency of the Pt ring.

**Computational methods.** Spin-unrestricted DFT computations were performed in the VASP code using the projector-augmented plane wave approach and GGA-PBE functional [1-3]. The plane-wave cutoff energy of 420 eV, which is ~1.6 times higher the suggested cutoff energy value in POTCAR, was adopted. The convergence criterions of energy and force were set to be 3 × 10^-5^ eV (smaller than the suggested default value of 10^-4^ eV) and 0.05 eV/Å (corresponding to a strict force criterion of 0.001 eV/ Å per atom), respectively. Specially, the force For MoX_2_ (X = S, Se, Te), both basal planes and Mo-edge sites were explored for the H_2_O_2_ production. Their basal plane slab was modelled as a (4×4×1) supercell of MoX_2_, while the Mo-edge slab was constructed with 50% X coverage and a periodicity of 3 Mo atoms (Figure S8). We noted that, similar to MoS_2_ [4], the Mo-edges of MoTe_2_ and MoSe_2_ also preferred to end with every three Mo atoms (3-Mo periodicity) (Figure S9). The vacuum space of 15 Å was included in all slabs to keep the image interaction negligible. The Brillouin zone was sampled with 3 × 3 × 1 and 1 × 3 × 1 *k*-points for basal plane and Mo-edge slabs, respectively. The free energies of species (G) were calculated by G = *E_DFT_ + E_ZPE_ − TS*, where *E_DFT_*, *E_ZPE_*, and *S* were ground state energy, zero-point energy, and entropy, respectively. The computational hydrogen electrode (CHE) model was used in determining the free-energy level in free-energy profile, and the theoretical overpotential (*η^t^*) of 2e ORR was deduced using the equation of *η^t^* = |ΔG_HOO*_/e − 4.22 V|. Since the activity descriptor Δ*G*_HOO*_ are established upon the nonconsideration of solvation effect [5-7], we did not include the solvation effect in determining the value of Δ*G*_HOO*_, which governs a resonable comparsion. Note that the consideration of solvation effect, which may bring a small stabilization (0.1~0.2 eV) on HOO*, should not change the reported active order in this work and previous studies [5-7].


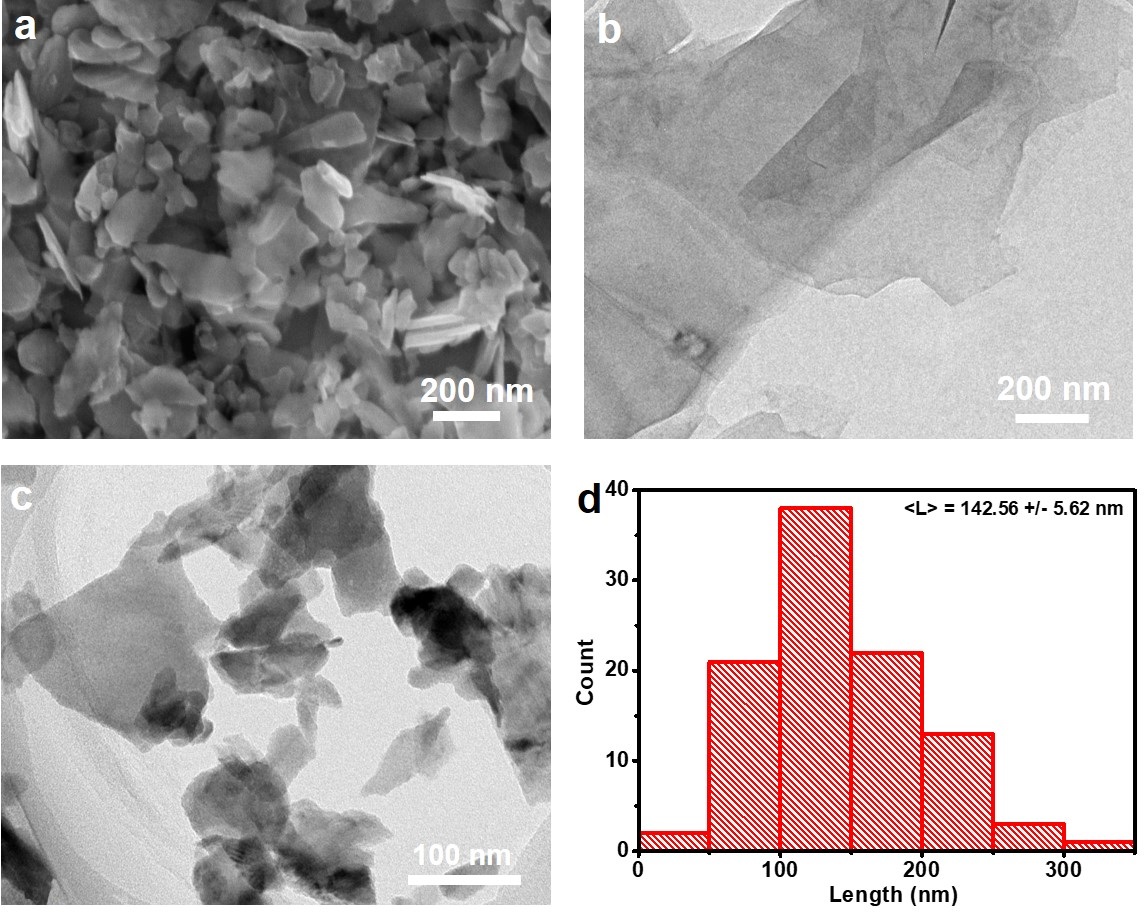


**Figure S1.** (a) SEM image and (b,c) low-magnification TEM images of MoTe_2_ nanosheets. (d) Histogram of the lateral size distribution of exfoliated MoTe_2_ nanosheets from TEM statistics.


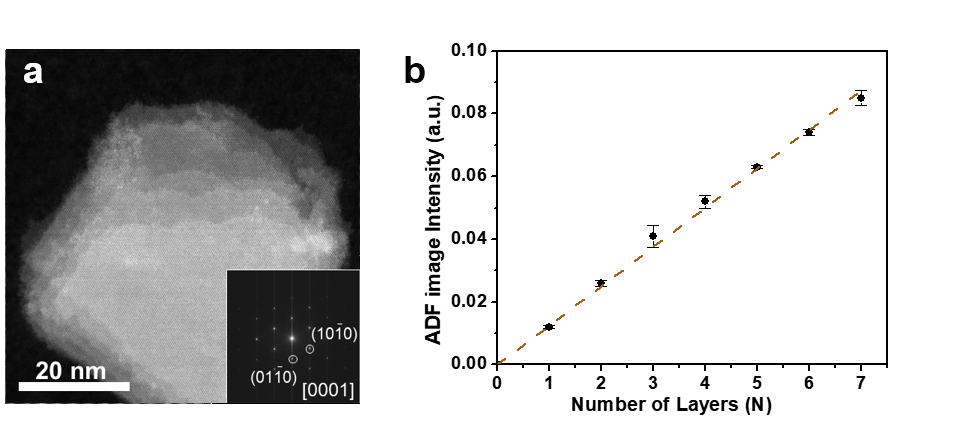


**Figure S2.** **Thickness measurement of MoTe_2_ nanoflakes via STEM imaging.** (a) Low-magnification STEM ADF image of a MoTe_2_ nanoflake containing 1 to 7 layers and (inset) corresponding FFT pattern. (b) Image intensity as a function of MoTe_2_ layer numbers from (a) showing the linear correlation between MoTe_2_ layers and image intensity for up to 7 layers.


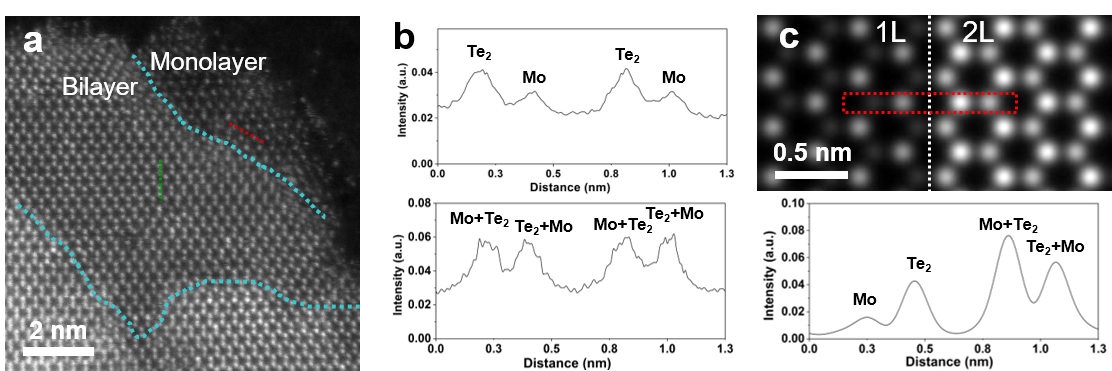


**Figure S3.** **STEM imaging for the determination of monolayer and bilayer.** (a) STEM ADF image of MoTe_2_ containing bilayer and monolayer regions. (b) Image intensity profiles along the two dashed lines in different regions: (top) monolayer region and (bottom) bilayer region. (c) (Top) simulated ADF image of 2H MoTe_2_ containing (left) monolayer and (right) bilayer; (bottom) corresponding image intensity profile along the dashed rectangle in the image above.


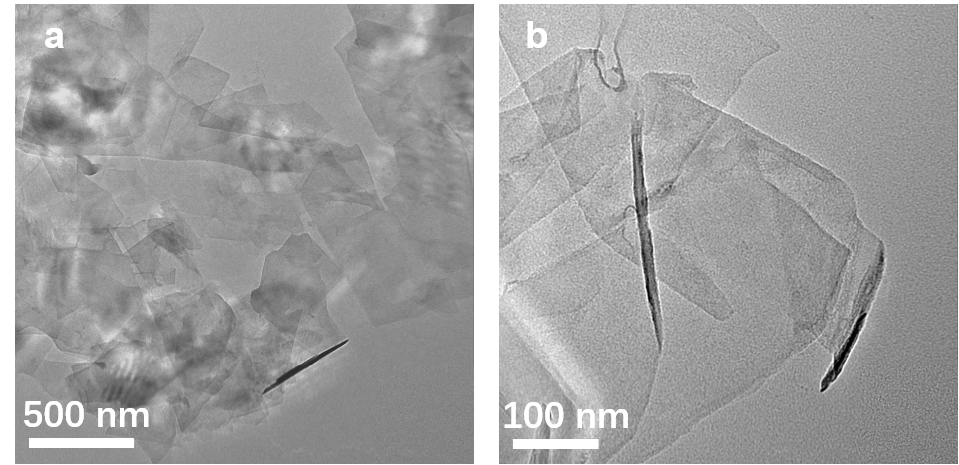


**Figure S4.** TEM images of exfoliated graphene nanosheets from LPE.


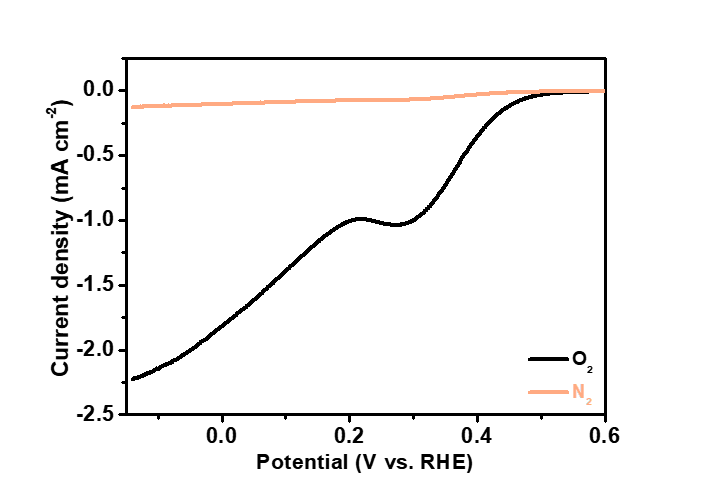


**Figure S5.** RRDE voltammograms of MoTe_2_ nanoflakes at 1600 rpm in O_2_- and N_2_-saturated 0.5 M H_2_SO_4_.


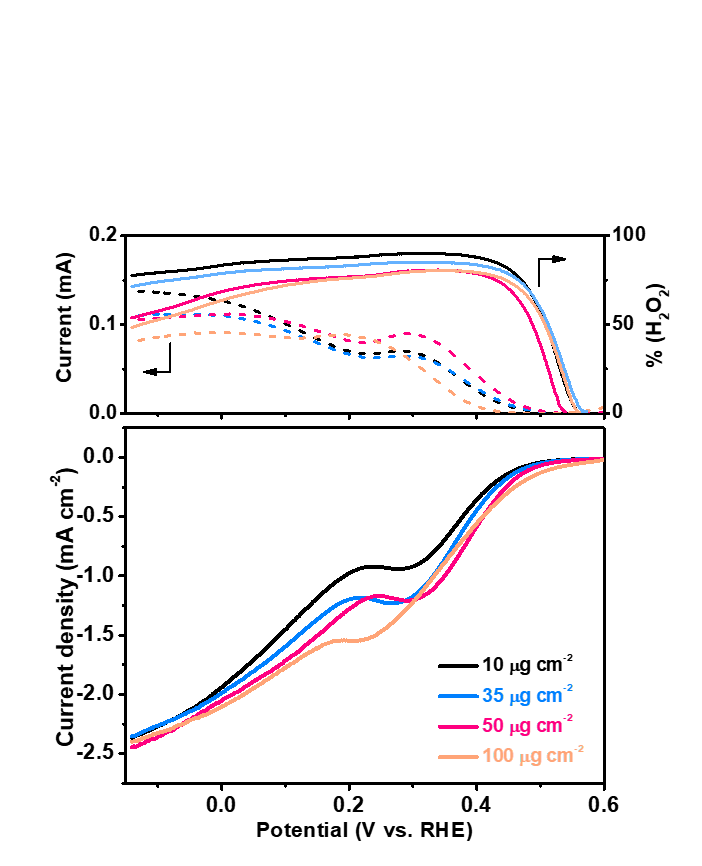


**Figure S6.** Polarization curves, ring currents and H_2_O_2_ percentage of MoTe_2_ nanoflakes at different areal loadings from 10 μg/cm^2^ to 100 μg/cm^2^.


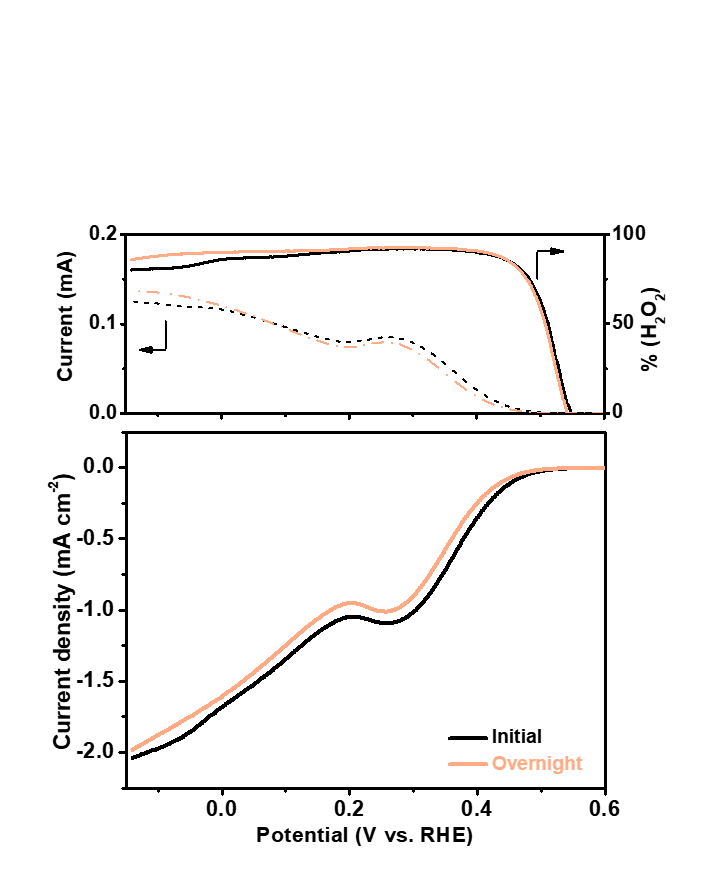


**Figure S7.** Change of the polarization curve, ring current and H_2_O_2_ percentage of MoTe_2_ nanoflakes after the overnight aging at the open circuit condition in O_2_-saturated 0.5 M H_2_SO_4_.


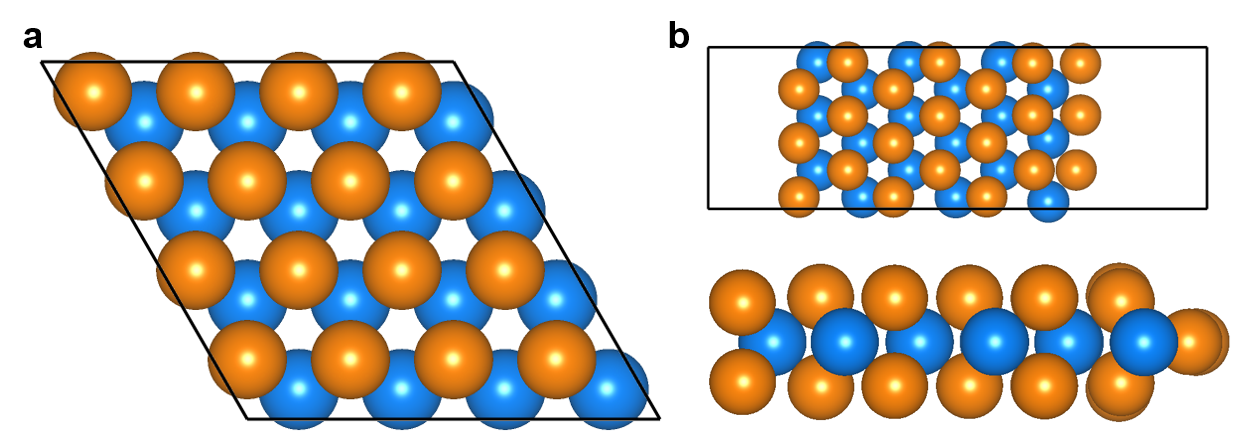


**Figure S8.** Optimized structure of 2H MoTe_2_: (a) basal plane and (b) Mo-edge sites. Mo and Te atoms are presented by blue and orange spheres, respectively.


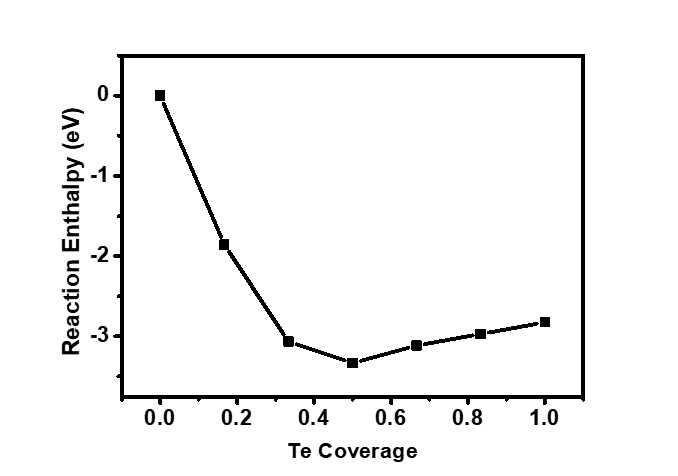


**Figure S9.** Calculated reaction enthalpy of the Te-terminated Mo-edge as a function of the Te coverage.

In order to estimate the most likely edge configuration, we calculated the reaction enthalpy (*E*_r_) of the Te-terminated Mo-edge. It was defined as *E*_r_ = (*E*_edge_ – *E*_bare_ − *N*_Te_*E*_Te_)/*N*_Te_, where *E*_edge_ was the total energy of an edge model, *E*_bare_ was the energy of the bare edge, *E*_Te_ was the energy of one Te atom, and *N*_Te_ was the number of Te atoms terminated in the edge. The lower the *E*_r_ value, the more stable the edge configuration was. As depicted above, 50% Te coverage processed the lowest *E*_r_, indicating that MoTe_2_ edges ideally preferred to be terminated with 50% Te coverage.


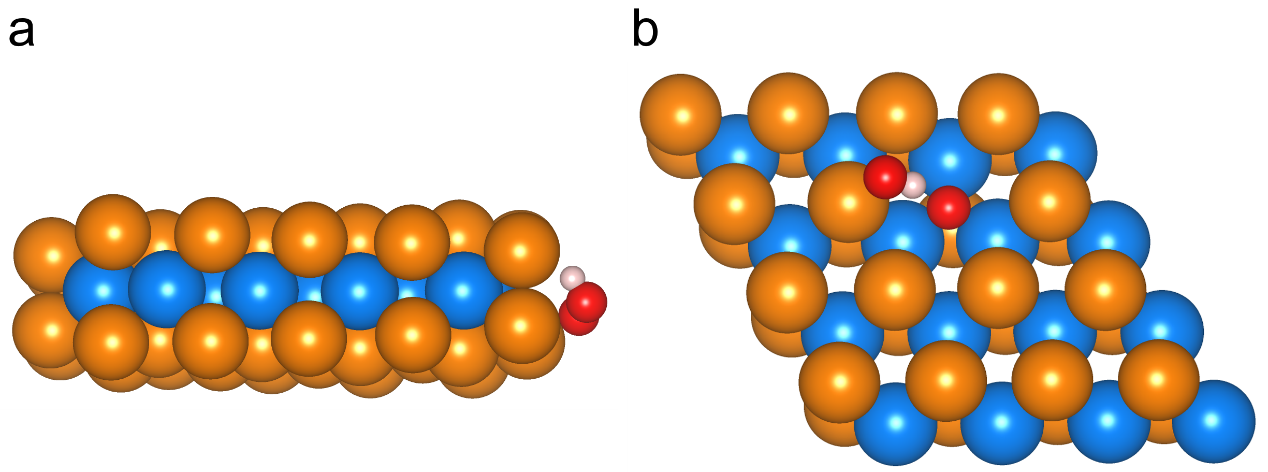


**Figure S10.** Optimized structures of O* and HO* from the dissociation of HOO* over a single Te vacancy on the basal plane of MoTe_2_.


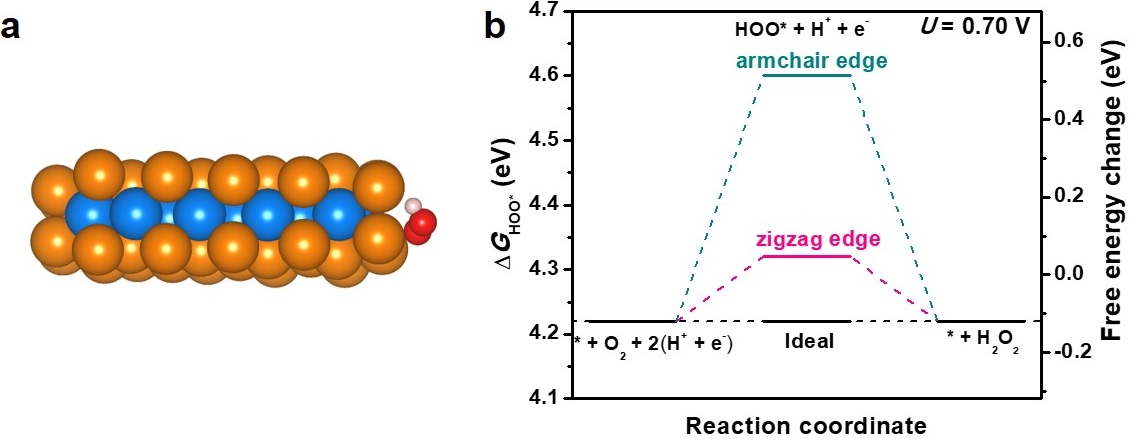


**Figure S11.** (a) Optimized structure of HOO* on the armchair edge. (b) ΔG_HOO*_ for 2e-ORR to H_2_O_2_ on armchair edge and egde on basal plane of MoTe_2_ at the equilibrium potential of U_O2/H2O2_ = 0.70 V and corresponding free-energy profiles.

**REFERENCES**

1. Kresse G, Furthmuller J. Efficient iterative schemes for ab initio total-energy calculations using a plane-wave basis set. *Phys Rev B* 1996; **54**: 11169-86.

2. Kresse G, Joubert D. From ultrasoft pseudopotentials to the projector augmented-wave method. *Phys Rev B* 1999; **59**: 1758-75.

3. Perdew JP, Burke K and Ernzerhof M. Generalized gradient approximation made simple. *Phys Rev Lett* 1997; **78**: 1396-.

4. Huang YF, Nielsen RJ and Goddard WA *et al*. The reaction mechanism with free energy barriers for electrochemical dihydrogen evolution on MoS_2_. *J Am Chem Soc* 2015; **137**: 6692-8.

5. Siahrostami S, Verdaguer-Casadevall A and Karamad M *et al*. Enabling direct H_2_O_2_ production through rational electrocatalyst design. *Nat Mater* 2013; **12**: 1137-43.

6. Verdaguer-Casadevall A, Deiana D and Karamad M *et al*. Trends in the electrochemical synthesis of H_2_O_2_: Enhancing activity and selectivity by electrocatalytic site engineering. *Nano Lett* 2014; **14**: 1603-8.

7. Lu ZY, Chen GX and Siahrostami S *et al*. High-efficiency oxygen reduction to hydrogen peroxide catalysed by oxidized carbon materials. *Nat Catal* 2018; **1**: 156-62.
